# Pharmacokinetics of dasatinib for Philadelphia-positive acute lymphocytic leukemia with acquired T315I mutation

**DOI:** 10.1186/1756-8722-5-23

**Published:** 2012-05-15

**Authors:** Naoto Takahashi, Masatomo Miura, Stuart A Scott, Takenori Niioka, Kenichi Sawada

**Affiliations:** 1 Dept. of Hematology Nephrology and Rheumatology, Akita Univ. Graduate School of Medicine, Akita, Japan; 2 Dept. of Pharmacy, Akita Univ. Hospital, Akita, Japan; 3 Dept. of Genetics and Genomic Sciences, Mount Sinai School of Medicine, New York, NY, USA

**Keywords:** Dasatinib, Ph positive acute lymphoid leukemia, T315I, Pharmacokinetics

## Abstract

**Background:**

The BCR-ABL T315I kinase domain mutation is insensitive to dasatinib therapy for Philadelphia-positive acute lymphoid leukemia (Ph + ALL) patients. Resistant T315I clone may be present prior to initiating dasatinib, which could expand under selective pressures during treatment. However, it is also possible that Ph + ALL patients newly acquire the T315I mutation during dasatinib therapy. Despite the potent inhibition of BCR-ABL kinase by dasatinib, little is known about the relationship between dasatinib pharmacokinetics and the emergence of kinase domain mutations in vivo.

**Methods:**

To determine whether plasma dasatinib pharmacokinetics influences the emergence of BCR-ABL mutations, we measured plasma dasatinib levels in 11 Ph + ALL patients undergoing dasatinib monotherapy.

**Results:**

Bone marrow relapse occurred in 5 of the 11 Ph + ALL patients (45%). Importantly, a T315I mutation was detected in 4 of the 5 relapsed patients, despite the absence of BCR-ABL mutations in any patient at baseline. The median plasma concentration at 2 hours (C_2h_), the median plasma maximum concentration (C_max_), and the median area under the observed plasma concentration-time curve from 0 to 4 hours (AUC_0-4_) were all significantly lower in patients with T315I than those without the mutation (C_2h_, 22.3 ng/mL vs. 111.6 ng/mL, *P* = 0.0242; C_max_, 43.8 ng/mL vs. 112.4 ng/mL, *P* = 0.0242; AUC_0-4_, 108.3 ng·h/mL vs. 268.3 ng·h/mL, *P* = 0.0061, respectively).

**Conclusions:**

These data indicate that the emergence of the T315I mutation among Ph + ALL patients treated with dasatinib is, in part, dependent on plasma dasatinib pharmacokinetics. Notably, these data also suggest that newly acquired BCR-ABL mutations may be inhibited by an increased exposure of dasatinib.

## Introduction

Dasatinib is a second-generation inhibitor of the BCR-ABL and SRC tyrosine kinases. In vitro, dasatinib inhibits the BCR-ABL kinase with 325-fold greater potency than imatinib [1]. In addition, it showed significant activity in Phase II studies in Philadelphia-positive acute lymphoid leukemia (Ph + ALL) patients who were resistant or intolerant to imatinib [2]. In pharmacokinetic studies, dasatinib exposure was shown to vary linearly and proportionally with dose. Maximum plasma concentration (C_max_) was observed 0.5 hours after a single oral administration, and the mean terminal elimination half-life (t 1/2) was <4 hours with rapid absorption.

Dasatinib has in vitro activity against all imatinib-resistant BCR-ABL mutations, with the notable exception of T315I [1]. For example, a previous study reported that 12 of 17 relapsed Ph + ALL patients acquired a T315I mutation during dasatinib therapy [3]. Additionally, there is little data on the relationship between plasma dasatinib concentration and outcome or adverse events, and no clinically relevant data to suggest that dose changes are necessary based on sex, age, or pharmacogenetic variation in dasatinib transporters. Moreover, little is known about the relationship between dasatinib pharmacokinetics and the emergence of BCR-ABL kinase domain mutations in vivo.

To determine whether plasma dasatinib pharmacokinetics influences BCR-ABL mutations , we used high-performance liquid chromatography (HPLC) to measure the plasma dasatinib concentrations in Ph + ALL patients undergoing dasatinib monotherapy.

## Methods

### Pharmacokinetic analyses

Plasma dasatinib concentrations prior to therapy and 1 to 4 hours after administration (C_0h_, C_1h_, C_2h_ and C_4h_) on day 7, which previously has been found to represent a stable period of dasatinib pharmacokinetics [4], were measured by HPLC. Pharmacokinetic analyses of dasatinib were performed using the standard non-compartmental method with WinNonlin (Pharsight Co., Mountain View, CA, version 4.0.1). The area under the observed plasma concentration-time curve from 0 to 4 hours (AUC_0-4_) was calculated using the linear trapezoidal rule. C_max_ was obtained directly from the profile.

### BCR-ABL Mutation analyses

Peripheral blood samples were obtained at baseline of dasatinib initiation and at bone marrow relapse. The bcr-abl fusion transcript was analyzed for mutations using direct sequencing.

### Statistical analyses

Statistical analyses were carried out using SPSS (SPSS Japan Inc., Tokyo, Japan, version 17.0) and the data are presented as medians (quartile 1-quartile 3). Differences in reported parameters between two patient groups were evaluated using the Mann-Whitney's U test. Time to event was measured from the date of dasatinib administration to the date of hematological relapse, date of death from any cause, or date of last molecular examination for patients who did not relapse. Event-free survival (EFS) was estimated using the Kaplan- Meier method. Values of *P* < 0.05 were considered significant.

### Study conduct

The study was conducted in accordance with the Declaration of Helsinki. Informed consent was obtained from all patients according to institutional guidelines. The study was approved by the Akita University Research Ethics Board.

## Results

Eleven patients with Ph + ALL received dasatinib monotherapy as an induction therapy according to previous study reported by Foà et al [3] (Table 1). The mean age was 59.0 years (range: 22–80 years), and 54.5% of the patients were male. No patient had severe hepatic or renal dysfunction. Median treatment duration of dasatinib monotherapy was 221 days (151.3-355.5 days).

**Table 1:** Patients and clinical outcomes with dasatinib monotherapy

**No**	**Age**	**Sex**	**Dasatinib dose**	**BM relapse**	**BCR-ABL mutation**	**EFS, day**	**Outcome / Event (cause of death)**
1	56	M	100 mg QD	No	No	624*	SCT in CMR
2	56	M	100 mg QD	No	No	149	dead (infection)
3	64	F	100 mg QD	No	No	318	CMR
4	68	M	100 mg QD	Yes	T315I	368	dead (PD)
5	67	F	100 mg QD	Yes	T315I	197	dead (PD)
6	22	F	100 mg QD	No	No	634*	SCT in CMR
7	77	M	50 mg BID	Yes	T315I	221	dead (PD)
8	63	F	50 mg QD	Yes	T315I	106	dead (PD)
9	80	F	100 mg QD	No	No	269	CMR
10	32	M	100 mg QD	No	No	158	CMR
11	78	M	100 mg QD	Yes	No	114	dead (PD)

BM, bone marrow; EFS, event-free survival; OS, overall survival; M, male; F, femal; QD, once a day; BID, twice a day; SCT, stem cell transplantation; CMR, complete molecular response; PD, progressive disease; * a date of SCT was evaluated as censoring for analysis of EFS.

There were no CNS leukemia or CNS relapses among any of the patients during dasatinib monotherapy and all patients achieved hematological remission; however, bone marrow relapse was observed in 5 of the 11 Ph + ALL patients (45%). Importantly, a T315I mutation was detected in 4 of the 5 relapsed patients by direct sequencing, despite the absence of BCR-ABL mutations in any patient at baseline. The median EFS for Ph + ALL patients with T315I was 197 days. In contrast, the median EFS for patients without T315I had not yet been reached, and the estimated 20-month survival rate was 70%.

**Figure 1:**
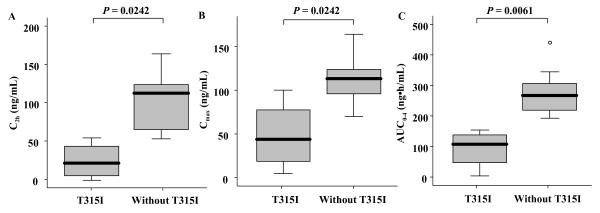
** Comparison of clinical and pharmacokinetic parameters of dasatinib in patients with and without a T315I mutation: (A) plasma concentration 2 h after dasatinib administration (C**_**2h**_**), (B) maximum plasma concentration after dasatinib administration (C**_**max**_**), and (C) area under the plasma concentration-time curve from 0 to 4 h (AUC**_**0-4h**_**).** Boxes represent interquartile ranges (IQR) with the median represented by bold horizontal lines. The ends of the whiskers (vertical lines) represent the smallest and largest values that are not outliers.

Table 2 summarizes the comparison between clinical characteristics and dasatinib pharmacokinetics in patients with and without T315I during dasatinib therapy. With the exception of platelet count, no correlations were detected with any clinical characteristics and T315I emergence; however, significant differences in plasma C_2h_, C_max_ and AUC_0-4h_ were detected between patients with T315I and those without (*P* = 0.0242, 0.0242, and 0.0061, respectively, Figure 1).

**Table 2:** Comparison of the clinical characteristics and dasatinib pharmacokinetics in patients with and without a T315I mutation

	**T315I (n = 4)**	**Without T315I (n = 7)**	***P-Value***
	**median (quartile 1- quartile 3)**	**median (quartile 1- quartile 3)**	
Sex ^†^ Female	2 (50.0)	3 (42.9)	0.6515
Age (year)	67 (64–72)	56 (38.5 - 71)	0.4121
Bodily weight (kg)	50.3 (48.3 - 60.1)	49.0 (44.2 - 52.8)	0.7879
Body surface area (m^2^)	1.49 (1.46 - 1.57)	1.45 (1.39 - 1.57)	0.6485
White blood cell (*10^3^/mm^3^)	2.4 (2.4 - 2.5)	3.7 (2.15 - 5.85)	0.3833
Red blood cell (*10^4^ mm^3^)	248 (241–282)	291 (238–316)	0.8333
Platelet (*10^4^ /mm^3^)	70 (50.5 - 77.5)	135 (117–167)	0.0167
Aspartate transaminase (IU/L)	32.5 (19.5 - 50)	33 (15.5 - 34)	0.5273
Alanine transaminase (IU/L)	23 (21–79)	23 (14–73.5)	0.7879
Serum albumin (g/dL)	4.3 (4.0 - 4.5)	3.9 (3.3 - 4.2)	0.2303
Total bilirubin (mg/dL)	0.4 (0.4 - 0.5)	0.4 (0.4 - 0.9)	0.9273
Serum creatinine (mg/dL)	0.6 (0.5 - 0.8)	0.6 (0.5 - 0.9)	0.7879
Single dose (mg)	100 (75–100)	100 (100–100)	0.7879
C_0h_ (ng/mL)	0.2 (0.1 - 0.4)	0.0 (0.0 - 0.0)	0.1091
C_1h_ (ng/mL)	13.2 (3.3 - 23.8)	49.0 (23.6 - 66.7)	0.0727
C_2h_ (ng/mL)	22.3 (5.8 - 43.8)	111.6 (65.1 - 122.8)	0.0242
C_4h_ (ng/mL)	34.6 (10.6 - 76.3)	69.7 (66.9 - 95.3)	0.1636
C_max_ (ng/mL)	43.8 (19.1 - 77.0)	112.4 (95.3 - 122.8)	0.0242
AUC_0-4h_ (ng·h/mL)	108.3 (49.2 - 139.2)	268.3 (220.0 - 307.3)	0.0061

^†^ Data presented as numbers (%) of patients;

C_nh_, plasma concentration at n hour after dasatinib administration;

C_max_, maximum plasma concentration less than 4 hours after dasatinib administration;

AUC_0-4h_, area under the plasma concentration-time curve from 0 to 4 hours.

## Discussion

The emergence of a BCR-ABL kinase domain mutation during dasatinib therapy, particularly T315I, is a significant concern that requires careful consideration of clinical management. The median time between dasatinib treatment initiation and T315I mutation detection in our study was 7.4 months (range, 3.5-12.3 months), which was similar to the 9.1 months reported in a previous retrospective study by Nicolini et al. [5].

Soverini et al. retrospectively analyzed Ph + ALL patients treated with dasatinib by cloning the BCR-ABL kinase domain in a bacterial vector and sequencing 200 independent clones per sample. Notably, T315I was detected at diagnosis in two of six patients who relapsed [6]. Although a T315I mutation could exist prior to dasatinib treatment, evidence from both clinical trials [3] and mouse models [7] indicates that in some cases it is newly acquired as a result of selective pressures during treatment. If a resistant T315I clone was present prior to dasatinib treatment, it would likely expand under selective pressures, and its prevention likely requires a novel agent targeting T315I leukemia. However, if a resistant T315I clone was newly acquired during proliferation due to insufficiency of dasatinib therapy, it may be possible to prevent acquired genetic tyrosine kinase mutations by an increased exposure of dasatinib.

An effective transient dasatinib level of 100 nM (approximately 50 ng/mL) is sufficient to inhibit the in vitro proliferation of most cell lines expressing imatinib-resistant BCR-ABL mutations, with the exception of T315I [8]. In vivo, the effective transient dasatinib level might represent a concentration sufficient to inhibit the proliferation of primary ALL cells prior to the emergence of T315I. Our data suggest that a plasma dasatinib concentration that inhibits proliferation of Ph + cells and induces apoptosis might inhibit the emergence of BCR-ABL mutations, including T315I, when a resistant T315I clone is not present prior to dasatinib treatment.

In conclusion, lower plasma concentrations of dasatinib were detected among Ph + ALL patients with T315I compared to those without the mutation. These data suggest that sufficient exposure of dasatinib may prohibit clonal evolution by adequately inhibiting BCR-ABL kinase. Future prospective studies with larger sample sizes of Ph + ALL patients are warranted to confirm these results.

## Competing interests

The authors declare no competing financial interests.

## Authors’ contributions

Contribution: NT designed and performed research and wrote the paper; MM performed the laboratory analysis; NT, MM, SAS and TN analyzed the data; NT and KS coordinated the study. All authors read and approved the final manuscript.
